# Der deutsche Fernbusmarkt — mittelfristig wieder auf ursprünglichem Niveau?

**DOI:** 10.1007/s10273-021-2964-8

**Published:** 2021-07-19

**Authors:** David Ennen, Janina Scheelhaase, Sven Maertens

**Affiliations:** 1 DLR - Institut für Flughafenwesen und Luftverkehr, Linder Höhe, 51147 Köln-Porz, Deutschland; 2 FW - Luftverkehrsforschung, DLR - Institut für Flughafenwesen und Luftverkehr, Linder Höhe, 51147 Köln-Porz, Deutschland

**Keywords:** L92, D57, I15

## Abstract

Der Fernbusverkehr ist zu Beginn der Pandemie fast vollständig zum Erliegen gekommen und nimmt seit Ende März 2021 seinen Betrieb wieder auf. Seit seiner Liberalisierung 2013 bietet der Fernbusverkehr nicht nur preiswerte Mobilität, sondern trägt auch zu Einkommen und Beschäftigung bei. Es ist zu erwarten, dass der Sektor wieder seine bisherige ökonomische Bedeutung erreicht, weil insbesondere Privatpersonen dieses Verkehrsmittel nutzen. Im Gegensatz zu Geschäftsreisen wird hier nach der Pandemie keine Verhaltensänderung erwartet.

## Abstract (en)

During the COVID-19 pandemic, long-distance bus transport in Germany came to an almost complete standstill. Since its liberalisation in 2013, long-distance bus transport has not only provided low-cost mobility, it has also contributed to income and employment in Germany. This article estimates the value added and employment created by the sector for the pre-COVID-19 year 2018. Our analysis suggests that the sector will reach pre-crisis levels relatively quickly as it mainly serves private travellers, especially VFR (visiting friends and relatives) travellers whose behavioural changes are less likely than those of business travellers.

Die Corona-Pandemie hat während der bisherigen Lockdowns zwischenzeitlich zu einer fast vollständigen Einstellung des Fernbusverkehrs in Deutschland geführt. Ende März 2021 nahm der führende Fernbusanbieter Flixbus den Betrieb wieder auf. Vor der Pandemie schien der Anfang 2013 liberalisierte Fernbusverkehr ein Plateau im Verkehrsmarkt erreicht zu haben. Nach einem anfangs starken Wettbewerb zwischen mehreren Anbietern setzte sich schließlich Flixbus nach einer Fusion mit Mein Fernbus Anfang 2015 als führendes Unternehmen durch, und die Verkehrszahlen stagnierten infolge der Konsolidierung zwischen 2015 und 2019 bei rund 21 Mio. bis 23 Mio. Passagier:innen pro Jahr (Statistisches Bundesamt, 2020b).

Wesentliche Gründe für die Politik, den Fernbusverkehr zu liberalisieren, waren die Schaffung einer preiswerten und emissionsarmen Alternative zum Auto sowie die Erhöhung des Wettbewerbsdrucks auf die Deutsche Bahn zur Verbesserung des Schienenpersonenfernverkehrs. Neben diesen positiven Effekten für private Haushalte und Umwelt sind durch die Liberalisierung auch neue Beschäftigung und Umsätze im Fernbussektor entstanden. Auf der Seite der Fernbusanbieter werden durch die Bereitstellung des Fernbusverkehrs Einkommen und Beschäftigung für Arbeitnehmer:innen und Geschäftsleute geschaffen. Diese ökonomischen Effekte werden nicht nur bei den Fernbusunternehmen selbst (direkte Effekte) generiert, sondern auch bei den Vorleistungsunternehmen in der Wertschöpfungskette, wie etwa Betreibern von Busbahnhöfen (indirekte Effekte). Zudem sind die Arbeitseinkommen der direkt und indirekt Beschäftigten für den Konsum mit weiteren, induzierten Effekten verbunden, denn die Herstellung von Konsumgütern und Dienstleistungen führt wiederum zu Einkommen und Beschäftigung. In einer modellgestützten Schätzung werden die durch den Fernbusverkehr geschaffenen Einkommen und Arbeitsplätze für 2018 herangezogen und anschließend mögliche Veränderungen im Fernbusmarkt nach der Corona-Pandemie abgeleitet.

## Fernbuslinienanbieter in Deutschland

Flixbus ist 2018 in Deutschland mit einem Anteil von 95,4 % an den inländischen und grenzüberschreitenden Fahrplankilometern der führende Anbieter von Fernbuslinienverkehren (IGES Institut, 2018a). Abbildung 1 stellt den Verkehrsanteil von Flixbus im Vergleich zu weiteren Anbietern wie Eurolines, der Deutsche-Bahn-Tochter IC Bus und RegioJet dar. Flixbus führt die Beförderungsleistung nicht selber durch, sondern beauftragt überwiegend mittelständische Busunternehmen mit dem ausführenden Passagiertransport, wobei Flixbus in den meisten Fällen als vertraglicher Beförderer dem Fahrgast gegenüber auftritt. Flixbus übernimmt dafür Linienplanung, Marketing, Vertrieb und Kundenservice. Einen Überblick über das

**Dr. David Ennen** ist wissenschaftlicher Mitarbeiter am Institut für Flughafenwesen und Luftverkehr des Deutschen Zentrums für Luft- und Raumfahrt (DLR) in Köln.

**Dr. Janina Scheelhaase** leitet dort die Abteilung Luftverkehrsökonomie.

**Dr. Sven Maertens** ist dort wissenschaftlicher Mitarbeiter.

Geschäftsmodell gibt Abbildung 2. Zwischen Flixbus und den beauftragten Beförderungsunternehmen bestehen Verträge, die eine Aufteilung der Ticketerlöse vorsehen. Medienberichten zufolge erhält Flixbus gemäß seines Standardvertrags 26 % und die Beförderungsunternehmen 74 % der Erlöse (Höfler, 2018). Flixbus bietet neben Deutschland in vielen weiteren Ländern Fernbusverkehre an, darunter europäische Länder wie Frankreich, Italien und Spanien, aber auch die USA. Für die Aktivitäten in den einzelnen Ländern hat Flixbus eigene Tochterunternehmen der Holding Flixmobility gegründet. Neben Busverkehren bietet Flixmobility mit ihrer Tochter Flixtrain seit 2017 auch Schienenpersonenfernverkehre auf wenigen Relationen in Deutschland an. Aufgrund des hohen Anteils am Fernbuslinienverkehr in Deutschland betrachten wir Flixbus im Folgenden als repräsentatives Unternehmen für die gesamte Branche und rechnen die geschätzten Größen auf den gesamten Fernbusverkehr anhand der erbrachten Fahrplankilometer hoch.

## Umsätze

Flixmobility weist in ihrem Geschäftsbericht (Flixmobility, 2020) den Umsatz für Deutschland aus, der neben den Umsätzen aus dem Fernbusgeschäft auch die aus dem Schienenpersonenfernverkehr umfasst. Mithilfe der Passagierzahlen von Flixbus und Flixtrain lässt sich der Umsatz von Flixbus in Deutschland schätzen. Die Passagierzahl von Flixbus ermitteln wir aus der Gesamtzahl der Fernbuspassagiere in Deutschland und dem Verkehrsanteil von Flixbus (Statistisches Bundesamt, 2020b; IGES Institut, 2018a). Die Passagierzahl von Flixtrain stammt aus einer Nachrichtenmeldung (Wacket, 2018). Der Umsatz der Beförderungsunternehmen ergibt sich gemäß der vermuteten Erlösaufteilung.

Der für den gesamten Fernbuslinienverkehr in Deutschland geschätzte Umsatz beträgt 272 Mio. Euro, wovon 201 Mio. Euro auf die reine Beförderung entfallen. Mit Verkehrszahlen (Statistisches Bundesamt, 2020a, 2020b) und unter der Annahme, dass die Hälfte der grenzüberschreitenden Passagiere und Passagierkilometer durch

Abbildung 1
**Verkehrsanteile der Fernbusanbieter nach Fahrplankilometern 2018**

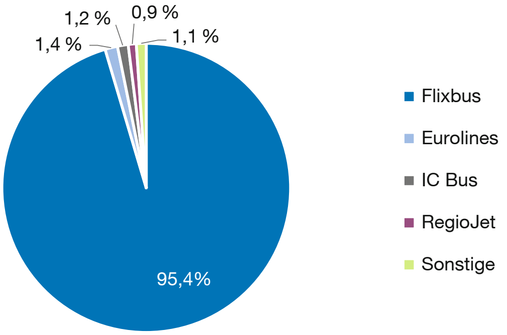

Quelle: eigene Darstellung in Anlehnung an IGES Institut (2018a).

Abbildung 2
**Geschäftsmodell von Flixbus**

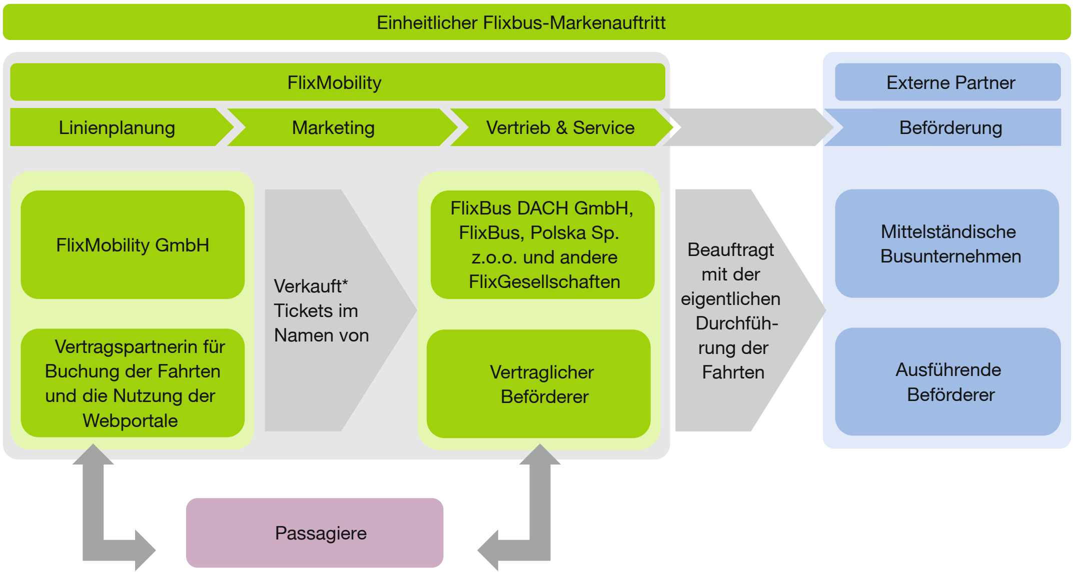

*in einzelnen Fällen auch Vermittlung von Tickets externer Beförderer.

Quelle: eigene Darstellung.

ausländische Busunternehmen befördert bzw. erbracht werden, ergibt sich ein durchschnittlicher Ticketpreis von 16,33 Euro (brutto) und ein durchschnittlicher Preis pro km von 5,8 Cent (brutto). Im Vergleich ermittelte das IGES-Institut durch Preisabfragen bei den Busanbietern einen durchschnittlichen Angebotspreis von 3,8 Cent pro km und einen Normalpreis von 10,7 Cent pro km für 2018 (IGES Institut, 2018a). Das Portal fernbusse.de berechnet auf Grundlage der über das Portal getätigten Buchungen einen Durchschnittspreis von 5,7 Cent pro km für 2017 (fernbusse.de, 2018). Der durchschnittliche Preis pro km im deutschen Schienenpersonenfernverkehr 2018 ist mit 12,6 Cent (brutto) gut doppelt so hoch (Bundesnetzagentur, 2019). Tabelle 1 fasst die Ergebnisse zusammen.

Tabelle 1
**Umsätze im Fernbuslinienverkehr 2018**

| Eckdaten | |
|---|---|
| Umsatz Insgesamt (Mio. Euro) | 272 |
| Umsatz Beförderung (Mio. Euro) | 201 |
| Durchschnittlicher Ticketpreis (brutto) (Euro) | 16,33 |
| Durchschnittlicher Preis pro km (brutto) (ct/km) | 5,8 |

Quelle: eigene Berechnungen.

## Vorleistungen

Die notwendigen Vorleistungen für die Beförderung schätzen wir mit einer Kostenrechnung für einen typischen Fernbuslinienbetrieb in Anlehnung an den Lastauto-Omnibus-Katalog 2018 (EuroTransportMedia, 2017). Der Katalog beinhaltet Kostenrechnungen für viele unterschiedliche Straßenschwerfahrzeuge und gilt als Referenz in der Branche. Das für unsere Berechnung gewählte Referenzfahrzeug ist der Reisebus Neoplan Cityliner C mit 55 Sitzplätzen, dessen Größe der tatsächlich eingesetzten Fahrzeuggröße von durchschnittlich 57 Sitzplätzen am nächsten kommt (Statistisches Bundesamt, 2020a). Abweichend vom Lastauto-Omnibus-Katalog rechnen wir mit der tatsächlichen durchschnittlichen Fahrleistung im Fernbuslinienverkehr von gut 194.000 km pro Jahr und Bus und einem durchschnittlichen Dieselkraftstoffpreis 2018 von 1,083 Euro/Liter (netto) (Bäumer et al., 2017; Mineralölwirtschaftsverband, 2020).

Die Kostenrechnungen im Lastauto-Omnibus-Katalog enthalten keine Fahrdienstkosten. Zur Berechnung der Fahrdienstkosten wird daher auf Bruttolohnangaben des Tarifvertrags für das private Omnibusgewerbe in Nordrhein-Westfalen zurückgegriffen, wobei für eine/n durchschnittliche/n Beschäftigte/n eine mittlere Qualifikation, fünf Jahre Betriebszugehörigkeit und zehn Krankheitstage im Jahr angenommen werden (Verband nordrhein-westfälischer Omnibusunternehmen et al., 2016). Bei der Berechnung der Fahrdienstkosten pro Buskilometer ist zu berücksichtigen, dass Flixbus bei Nachtfahrten grundsätzlich zwei Fahrende einsetzt und gemäß einer Umfrage unter Fernbuspassagier:innen 16 % der Fahrten Nachtfahrten waren (Bruns et al., 2018). Zudem sind Betriebsfahrten notwendig, bei denen keine Beförderungsleistung erbracht wird (z. B. vom Betriebshof zur ersten Haltestelle). Der Dienstplanwirkungsgrad, der das Verhältnis von Dienstzeit zu Fahrzeit angibt, ist daher kleiner als eins und wird Rieken et al. (2015) folgend mit 75 % angenommen. Die zugrunde gelegte durchschnittliche Fahrplangeschwindigkeit beträgt gemäß einer Stichprobe 67,7 km/h (Bruns et al., 2018).

Die errechneten Beförderungskosten sind in Tabelle 2 aufgeführt, und Abbildung 2 veranschaulicht deren Anteil an den Gesamtkosten. Ein Großteil der Kosten sind mit 46 % Personalkosten, die auf Fahrdienst, Reparatur, Wartung, Pflege und Verwaltung entfallen. Die nächst größeren Kostenpositionen sind Kraftstoffkosten mit 22 % und Fahrzeugabschreibungen mit 19 %. Insgesamt ergeben sich Kosten von 1,57 Euro pro Buskilometer. Becker et al. (2008) berechnen in Anlehnung an Leuthardt (1998) die Kosten im Fernbuslinienverkehr und erhalten Kosten von 1,62 Euro pro Buskilometer für 2008, was ca. 1,76 Euro in Preisen von 2018 entspricht (Statistisches Bundesamt, 2018).[1] Hierbei ist jedoch anzumerken, dass Becker et al. (2008) für 2008 mit einem deutlich höheren Dieselpreis von 1,40 Euro/Liter und einer niedrigeren Jahresfahrleistung von 160.000 km rechnen.

Als weiteren Vergleich lässt sich der Umsatz pro Buskilometer errechnen, den die Beförderungsunternehmen im Mittel von Flixbus erhalten. Gemäß unseres geschätzten Beförderungsumsatzes und der inländischen Verkehrsleistung in Buskilometer (Statistisches Bundesamt, 2020a) ergibt sich ein Umsatz pro Buskilometer von 1,42 Euro. Dieses Ergebnis ist knapp 10 % geringer als die geschätzten Kosten, was darauf hindeutet, dass die Beförderungsunternehmen entweder kaum profitabel für Flixbus fahren können oder unsere Rechnung die tatsächlichen Kosten geringfügig überschätzt. Nach Medienberichten haben einige Busunternehmen ihre Verträge mit Flixbus nach Verlusten wieder gekündigt. Zudem sind bei einigen Busunternehmen hohe Verbindlichkeiten gegenüber Flixbus aufgelaufen. Diese waren dadurch bedingt, dass Flixbus eine Zahlung von 1,10 Euro pro Buskilometer garantiert, jedoch den durch Erlöse ungedeckten Anteil lediglich als Darlehen vergibt (Burgschat und Fuchs, 2016). Für die weitere Berechnung nehmen wir an, dass die Beförderungsumsätze genau kostendeckend sind und sich diese gemäß den ermittelten

1 Gemäß dem Erzeugerpreisindex für Straßengüterverkehr.

Tabelle 2
**Beförderungskosten im Fernbuslinienverkehr 2018**

| | Pro Bus-km (in ct) | Anteil (in %) |
|---|---|---|
| Personalkosten | 72,4 | 46,1 |
| Fahrdienst | 43,1 | 27,4 |
| Reparatur, Wartung | 24,2 | 15,4 |
| Verwaltung | 5,2 | 3,3 |
| Fahrzeugabschreibung | 29,9 | 19,0 |
| Kraftstoffkosten | 34,7 | 22,0 |
| Verbrauch (ohne Energiesteuer) | 19,6 | 12,5 |
| Energiesteuer | 15,1 | 9,6 |
| Materialkosten | 0,6 | 2,9 |
| Reifenkosten | 10,2 | 2,5 |
| Schmierstoffkosten | 4,6 | 0,4 |
| Kapitalverzinsung | 4,5 | 6,5 |
| Versicherungskosten | 4,0 | 2,9 |
| Sonstige Kosten | 0,9 | 0,6 |
| Kfz-Steuer | 0,3 | 0,2 |
| Stellplatzkosten | 0,6 | 0,4 |
| Gesamtkosten | 157,2 | 100,0 |

Kosten für Reparatur, Verwaltung und Wartung vollständig den Personalkosten zugeschlagen.

Quelle: eigene Berechnungen.

Abbildung 3
**Beförderungskosten im Fernbuslinienverkehr 2018**

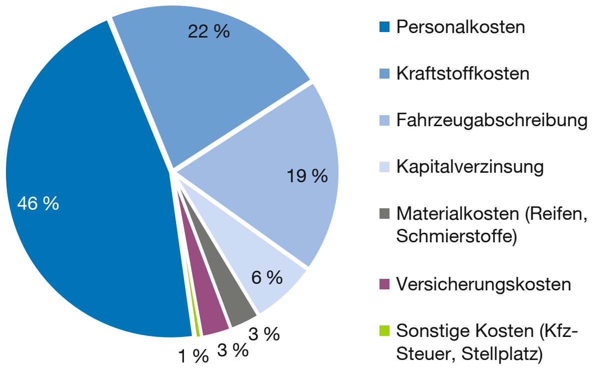

Quelle: eigene Berechnungen.

Anteilen auf die einzelnen Kostenpositionen aufteilen. Zu den Vorleistungen zählen dabei die Ausgaben für Kraftstoff, Reifen, Schmierstoff, Versicherung und Stellplatzmiete.

## Direkte Bruttowertschöpfung und Beschäftigung

Die Bruttowertschöpfung ergibt sich grundsätzlich als Produktionswert abzüglich der bezogenen Vorleistungen. Für die von Flixbus selbst erbrachte Linienplanungs-, Vertriebs- und Serviceleistung im Fernbusgeschäft (im Folgenden vereinfacht als Vertrieb bezeichnet) greifen wir auf den Geschäftsbericht von Flixmobility zurück und berechnen durch Abzug der Vorleistungen vom Umsatz den Bruttowertschöpfungsanteil. Damit und mit dem bereits geschätzten Umsatz des gesamten Fernbuslinienverkehrs in Deutschland ergibt sich die durch den Vertrieb geschaffene Bruttowertschöpfung. Die Bruttowertschöpfung in der Beförderung berechnen wir analog mit dem bereits ermittelten Bruttowertschöpfungsanteil am Beförderungsumsatz.

Die direkte Beschäftigung setzt sich aus der Beschäftigung im Vertrieb und in der eigentlichen Beförderung zusammen. Flixmobility gibt die weltweite Beschäftigtenzahl in ihrem Geschäftsbericht an, die über die weltweite Passagierzahl (Flixbus, 2019) und die Passagierzahl von Flixbus in Deutschland dem deutschen Fernbusgeschäft zugerechnet werden kann. Die Beschäftigung in der Beförderung schätzen wir analog mithilfe von Angaben von Flixbus über die weltweite Zahl von Fahrenden, die die ausführenden Beförderungsunternehmen auf Flixbuslinien einsetzen (Wembridge, 2019). Neben den Beschäftigten im Fahrdienst sind bei den Beförderungsunternehmen noch die Beschäftigten in Technik und Verwaltung zu berücksichtigten. Durch Zahlen des Statistischen Bundesamts (2020a), wie viele Beschäftigte in Technik und Verwaltung in SPNV- und Busunternehmen auf eine/n Beschäftigte/n im Fahrdienst kommen, schätzen wir die Gesamtbeschäftigung in der Fernbusbeförderung. Die sich ergebene direkte Bruttowertschöpfung beträgt 169 Mio. Euro und die direkte Beschäftigung knapp 5.000 Arbeitsplätze (vgl. Tabelle 3). Ein Großteil der ökonomischen Effekte entsteht mit über 85 % bei der Erbringung der Beförderungsleistung und nur ein kleiner Teil von unter 15 % durch die Vertriebsleistung (Linienplanung, Vertrieb, Service).

## Gesamte Bruttowertschöpfung und Beschäftigung

Mit der Input-Output-Rechnung (Leontief, 1986) und der Input-Output-Tabelle des Statistischen Bundesamts für 2017, die auf empirischen Daten basiert, berechnen wir die indirekten und induzierten Effekte des Fernbuslinienverkehrs. Dieser ist Teil des stark aggregierten Wirtschaftszweigs „Landverkehrsleistungen und Transportleistungen in Rohrfernleitungen" der Input-Output-Tabelle, dessen Vorleistungsbezüge die Produktionsstruktur des Fernbuslinienverkehrs nur unzureichend repräsentieren. Aufbauend auf den bereits ermittelten Informationen über Umsätze und Vorleistungsbezüge in Vertrieb und Beförderung schätzen wir einen neuen Wirtschaftszweig „Fernbusverkehrsleistungen" in der Input-Output-Tabelle. Hierbei ordnen wir die bezogenen Vorleistungen den einzelnen Wirtschaftszweigen zu und übernehmen diese sowie Arbeitnehmerentgelte, Abschreibungen, Zinsen, Betriebsüber-

Tabelle 3
**Direkte Bruttowertschöpfung und Beschäftigung im Fernbuslinienverkehr 2018**

| Direkte Bruttowertschöpfung | in Mio. Euro | Anteil (in %) |
|---|---|---|
| Linienplanung, Vertrieb, Service | 25 | 15 |
| Beförderung | 144 | 85 |
| Insgesamt | 169 | 100 |
| Direkte Beschäftigung | Arbeitsplätze | Anteil (in %) |
| Linienplanung, Vertrieb, Service | 650 | 13 |
| Beförderung | 4.318 | 87 |
| Fahrdienst | 3.079 | 62 |
| Technik | 655 | 13 |
| Verwaltung | 583 | 12 |
| Insgesamt | 4.968 | 100 |

Quelle: eigene Berechnungen.

Abbildung 4
**Bruttowertschöpfung und Beschäftigung durch den Fernbuslinienverkehr 2018**

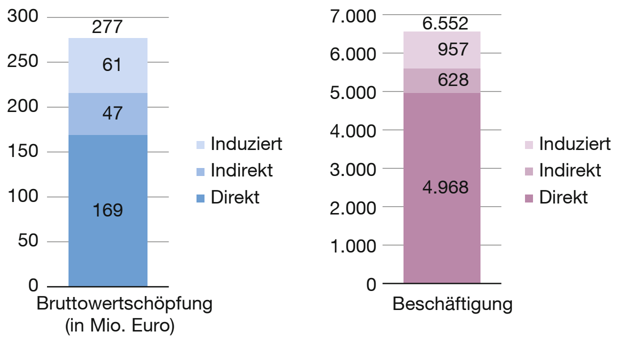

Quelle: eigene Berechnungen.

schüsse und Steuern in die entsprechenden Zellen der Input-Output-Tabelle. Die indirekten Effekte sind ein unmittelbares Ergebnis der Input-Output-Rechnung. Für die induzierten Effekte ermitteln wir zunächst das Arbeitseinkommen, das direkt und indirekt durch den Fernbusverkehr entsteht, und leiten daraus die zusätzliche Konsumnachfrage ab, um dann mit der Input-Output-Rechnung die induzierte wirtschaftliche Aktivität bei Konsumgüterproduzierenden und Dienstleistenden zu berechnen.

Die direkte, indirekte und induzierte Bruttowertschöpfung und Beschäftigung sind in Abbildung 4 dargestellt. Insgesamt generiert der Fernbuslinienverkehr schätzungsweise eine Bruttowertschöpfung von 277 Mio. Euro für die deutsche Volkswirtschaft und bietet Beschäftigung für gut 6.500 Erwerbstätige. Wie in Abbildung 4 zu erkennen ist, machen die direkte Bruttowertschöpfung mit 169 Mio. Euro und die direkte Beschäftigung mit knapp 5.000 Erwerbstätigen den Großteil des ökonomischen Gesamteffekts aus. Die indirekten Effekte fallen dagegen verhältnismäßig klein aus. Wesentlicher Grund hierfür ist, dass der Anteil bezogener Vorleistungen des Fernbuslinienverkehrs relativ gering ist und diese teilweise nur eine geringe Wertschöpfungstiefe im Inland aufweisen. Zu den nennenswerten Vorleistungen zählen Kraftstoff der Mineralölindustrie, die Bereitstellung der Busbahnhöfe, Werbung und Versicherungsleistungen. Der in Deutschland verbrauchte Kraftstoff wird zum Teil im Ausland produziert, und die inländischen Kraftstoffhersteller sind zum allergrößten Teil auf Erdölimporte aus dem Ausland angewiesen. Ein erheblicher Teil der Wertschöpfungskette in der Kraftstoffproduktion entfällt daher auf das Ausland. Darüber hinaus ist Raffinierung von Erdöl nur mit einem geringen Arbeitseinsatz verbunden, was die niedrigen indirekten Beschäftigungseffekte erklärt.

## Deutscher Fernbusmarkt nach der Pandemie

Wir schätzen, dass der Fernbussektor in Deutschland nach der COVID-19-Pandemie in den kommenden Jahren wieder etwa sein Niveau von 2019 erreichen wird. Wichtig hierfür wird sein, dass nationale und internationale Reisebeschränkungen sowie Kontaktbeschränkungen über die zulässige Zahl von Haushalten wieder aufgehoben oder zumindest stark gelockert werden (z. B. durch Ausnahmen für Geimpfte und Genesene). Diese Einschätzung stützt sich auf folgende Beobachtungen:

*Erstens* zeichnet sich der Fernbusverkehr z. B. im Gegensatz zum Luftverkehr durch einen hohen Anteil nationaler Verkehre aus, die von internationalen Reisebeschränkungen oder Quarantänebestimmungen nicht betroffen sind. Der Inlandsverkehr machte in Passagierzahlen mehr als 70 % des deutschen Fernbusverkehrs 2019 aus (Statistisches Bundesamt, 2020a). *Zweitens* sind ein Großteil der Fernbuspassagiere Privatreisende, die Freunde oder Verwandte besuchen oder in den Urlaub fahren. Mögliche Verhaltensänderungen, die auch nach der Pandemie bestehen bleiben könnten, wie z. B. die vermehrte Nutzung von Videotelefonie, betreffen vorwiegend den Geschäftsreiseverkehr, der jedoch nur ca. 5 % des Fernbusverkehrs ausmacht (IGES Institut, 2018b). *Drittens* ist der durchschnittliche Fernbusfahrgast mit einem mittleren Alter von rund 35 Jahren relativ jung (IGES Institut, 2018b) und fürchtet vermutlich weniger die Folgen einer Infektion, sodass die Reisebereitschaft weniger stark betroffen sein sollte.

## Schlussfolgerungen

Der Fernbuslinienverkehr ermöglicht nicht nur preiswerte und emissionsarme Mobilität, sondern schafft auch

Einkommen und Beschäftigung. Die Einkommens- und Beschäftigungseffekte des Fernbuslinienverkehrs in Deutschland umfassen direkte Effekte bei den Verkehrsunternehmen, indirekte Effekte bei Vorleistern wie dem Mineralölsektor oder Busbahnhofbetreibenden und induzierte Effekte bei Konsumgüterproduzenten und Dienstleistenden durch Konsumausgaben der direkt und indirekt Beschäftigten. Unseren modellgestützten Berechnungen zufolge wurden 2018 eine Bruttowertschöpfung in Höhe von 277 Mio. Euro generiert und etwa 6.500 Arbeitsplätze durch den Fernbuslinienverkehr in Deutschland ermöglicht. Die errechneten Effekte dürfen jedoch nicht vollständig als zusätzliche Einkommen und Beschäftigung als Folge der Liberalisierung des Fernbusverkehrs verstanden werden. Durch neue ökonomische Aktivität in einer Volkswirtschaft werden bei hohem Beschäftigungsstand auch immer bestehende Aktivitäten verdrängt.

Die COVID-19-Pandemie hat zum nahezu vollständigen Erliegen des Fernbusverkehrs in Deutschland geführt. Ende März 2021 nahm schließlich der größte deutsche Fernbusanbieter Flixbus den Betrieb wieder auf. Unseren Schätzungen zufolge wird der Fernbussektor in Deutschland in den kommenden Jahren wieder etwa sein Vor-Corona-Niveau erreichen. Denn gerade die private Nachfrage nach Besuchs- und Urlaubsverkehren dürfte im Nachgang zur Pandemie wenig Änderungen unterliegen. Wichtig hierfür wird sein, dass die staatlicherseits eingeführten Reise- und Kontaktbeschränkungen aufgehoben oder zumindest stark gelockert werden.
